# Study on Machine Tool Positioning Uncertainty Due to Volumetric Verification

**DOI:** 10.3390/s19132847

**Published:** 2019-06-26

**Authors:** Sergio Aguado, Pablo Pérez, José Antonio Albajez, Jorge Santolaria, Jesús Velazquez

**Affiliations:** 1 Centro Universitario de la Defensa, Academia General Militar, Ctra. Huesca S/N, 50090 Zaragoza, Spain; 2 Design and Manufacturing Engineering Department, University of Zaragoza, 50018 Zaragoza, Spain

**Keywords:** Monte Carlo method, machine tool uncertainty, calibration, measurement in process, traceability

## Abstract

Volumetric verification is based on the machine tool (MT) kinematic model, along with its geometric errors. Although users often ignore the uncertainty of verification, the use of the MT as a traceable measurement system in the manufacturing process has increased the need for professionals to be aware of it. This paper presents an improvement in the MT kinematic model, introducing in it the influence of verification uncertainty sources. These sources have been classified into four groups: the MT, the measurement system itself, the measurement strategy, and the optimization strategy. As the developed model exhibits non-linear behavior, the Monte Carlo method was used to determine the influence of the measurement system on verification uncertainty using synthetic tests. In this manner, an improved estimation of the MT uncertainty can be obtained. Therefore, if the MT is used as a traceable measurement system, its accuracy should not be higher than the laser tracker (LT) verification influence. It hence shows the importance of LT influence.

## 1. Introduction

The accuracy of the machined parts and the feedback information of the manufacturing process are two of the most critical considerations for any manufacturer. These considerations are the result of a number of influences and sources of error, such as the cutting conditions, the part type, and the machine tool (MT) characteristics.

These errors can be classified as either random or systematic errors, including quasi-static and dynamic ones. Quasi-static errors are divided into geometric, kinematic, and thermal errors. Geometric errors are the result of structural elements and they reduce the positioning accuracy of the MT. The direction of motion generated by joints, couplings, gears, and stiffness errors cause deformations and generate kinematic errors. Thermal errors are the result of temperature gradients in the structure of a machine or part, which generate dimensional changes that affect accuracy. Dynamic errors are caused by sources such as the vibration of the MT structure, the spindle movement errors, or the software. Therefore, the accuracy of the MT is provided by random and systematic errors, which are not compensated after verification processes.

**Figure 1:**
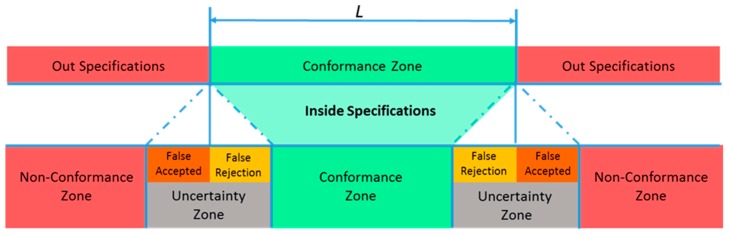
Influence of uncertainty on the conformance zone.

Uncertainty should be calculated to evaluate whether additional manufacturing tolerances can be achieved. When an MT is used as a measurement system with a probe to determine part dimensions, the MT measurement uncertainty determines the conformance zone. Figure 1, whose upper bar shows specifications and lower bar the influence of uncertainty, shows that false acceptance could occur if the uncertainty interval is outside the tolerance zone, but the measurement remains within the tolerance limits. However, false rejection could occur when the measured value is within the tolerance zone, but the uncertainty is higher. If uncertainty becomes minimized, the conformance zone is increased, thus reducing false acceptance and rejection. In 2006, Forbes illustrated conformance and non-conformance zones based on uncertainty values [1].

In 2000, Ahn et al. [2] analyzed the influence of geometric error variances in volumetric verification. In 2009, Bringmann et al. [3] identified that there is little correlation between the employed measurement system and the MT configuration, thereby improving the selection of the instrument and the test method to be used for geometric verification. In 2011, Andolfatto et al. [4] presented a method to evaluate contributions for the identification of linking errors of a five-axis MT, which was characteristic of the measurement system used. In 2015, Liu et al. analyzed the influence of laser tracker (LT) uncertainty on the volumetric verification under environmental conditions [5].

Although users often ignore the uncertainty of verification, the use of the MT as a traceable measurement system in the manufacturing process [6] has increased the need for users to be aware of it. In this paper, the sources of uncertainty that affect MT volumetric verification are classified and analyzed into four groups: the MT, the measurement system itself, the measurement strategy, and the optimization strategy. All uncertainty sources have been modelled and introduced to the MT kinematic chain to obtain their joint influence using the Monte Carlo method to determine how different LT locations affect measured points. The same method has also been used to estimate the influence of measurement uncertainty on MT verification by analyzing its probability distribution function (PDF). In this manner, the accuracy of the machining parts and a positioning accuracy better than the LT uncertainty influence cannot be ensured.

## 2. Materials and Methods

Volumetric verification faces MT error characterization minimizing their joint influence of its geometric errors. The relation between the position of the tool tip and that of the workpiece is obtained through the MT equation of movement. To calculate this equation, first the MT type must be determined. Second, the MT kinematic chain, including the MT geometric errors and the LT location, is modelled mathematically. Finally, the differences between measured and nominal points are optimized to obtain geometric errors. As the MT kinematic model is different on each MT. Section 2.1 presents the MT to analyze within its sequence of movements. Meanwhile, Section 2.2 shows the general verification procedure using as example the MT presented in Section 2.1.

### 2.1. Materials

Machine types are classified according to the movement of the part, the tool, and the axes of motion (kinematic chain). In this paper, a three-axis MT model ANAYAK VH1800 is studied, where the X axis moves with the part and the Y–Z axes move with the tool; hence, the MT is of the XFYZ type, where F represents the bed of the machine, the letter on the left of F represents the axis, which moves the workpiece, and letters on the right represent the axes, which move the tool.

Matlab software was used to model the kinematic chain and to run the optimization and the simulations.

### 2.2. Methodology

**Figure 2:**
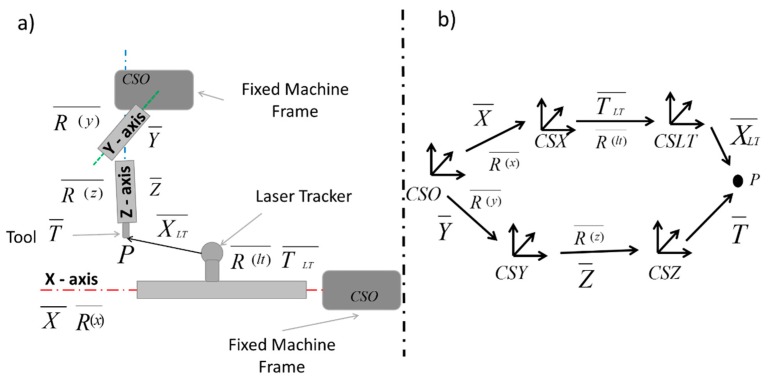
(**a**) Layout of measuring system in a MT with XFYZ configuration. (**b**) Kinematic scheme of a MT with XFYZ configuration.

The kinematic chain is defined through structural components of the machine and symbolizes the flow of the movements of the machine. Slocum [7] and Duffie et al. [8] presented a procedure based on homogeneous transformation matrices between axes (assuming the rigid-body hypothesis) and geometric errors. Wenjie [9] used these matrices as a systematic approach for the modelling of the geometric error of MTs; Rahman et al. [10] measured intra-axis and inter-axis errors in the kinematic model of the MT using a touch trigger probe as the measurement system, and Khan et al. [11] developed a systematic method to obtain the kinematic model of an MT with five axes of movement. Aguado et al. [12,13] introduced an LT measurement system on the MT model, as shown in Figure 2, where each axis is represented by a coordinate system, which is modelled by a rotational matrix and a translational vector.

Figure 2a schematically shows the complete system XFYZ MT + LT. Figure 2b shows the two paths that link the fixed machine frame to contact point P. In an ideal MT, both chains should provide the same point P; however, in actuality, each chain has a different error, e_i_. The error for each point i is the difference between the real tool-tip position P_r_ and the nominal position P_n_, with *r* subscript for real coordinates and *n* for nominal values, introduced by the numerical control (Equation (1)).

(1) $$e_{i}\;\left(e_{i,x},e_{i,y},\;e_{i,z}\right)=P_{i,r}\;\left(x_{i,r},y_{i,r},\;z_{i,r}\right)-P_{i,n}\;\left(x_{i,n},y_{i,n},\;z_{i,n}\right)$$

To link the geometric errors of the MT with the measured errors, it is necessary to solve the mathematical problem of equating both chains (Figure 2b); this may be achieved by providing the equation of motion of the MT (Equation (2)) where bar letters represent vectors or matrices that provide laser tracker coordinates from MT nominal positions taking into consideration the MT architecture.

(2) $$\bar{X_{LT}}=\;\bar{R_{LT}^{-1}}\;\left(\bar{R_{X}^{-1}}\;\left(\bar{R_{y}}\;\left(\bar{R_{z}}\cdot \;\bar{T}+\;\bar{Z}\right)+\;\bar{Y}-\bar{X}\right)-\bar{T_{LT}}\right)$$

**Figure 3:**
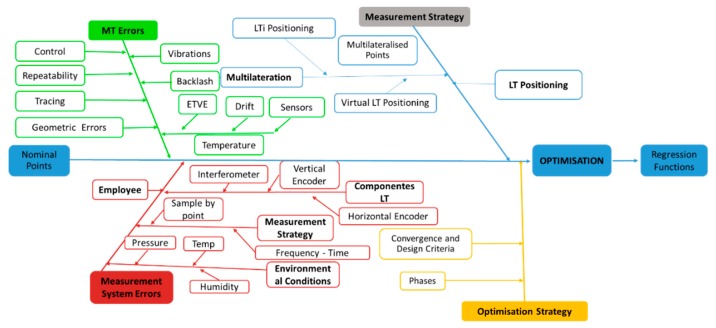
Uncertainty sources in MT verification when it is used as a measurement system. LT is laser tracker.

The error of a point is influenced by different uncertainty sources, which also affect the verification result. Uncertainty sources, which affect the volumetric verification based on LT, have been divided into the following (Figure 3):

1. Uncertainty related to the MT: repeatability, geometric errors, environmental conditions, control of the MT, etc.
2. Uncertainty related to the employed measurement system (LT): environmental conditions, uncertainty of measurement components, and measurement design.
3. Uncertainty related to the measurement strategy: LT positioning and techniques to improve measurement accuracy as multilateration.
4. Uncertainty related to the optimization strategy: values of converging criteria used in the identification process, sequence of identification (optimization phases), and the approximation function used.

#### 2.2.1. Uncertainty Related to the MT

Within all MT uncertainty sources that affect the accuracy of measurement, such as repeatability, control, or backlash, the most relevant are the temperature and geometric errors [12,13].

To estimate the influence of temperature on the MT verification, several temperature sensors should be placed along the MT structural elements, such as the column, bed, and ram of the machine (Equation (3)). Sensors have an uncertainty $u_{device}$ associated with a calibration uncertainty $u_{calibration}$; their influence was modelled as a Type B uncertainty using their calibration certificate [14]:

(3) $$u_{device}=\;\frac{u_{calibration}}{k}.$$

During verification, the spindle does not rotate, and the machine moves slowly. Therefore, if the MT was previously moving unloaded during the period of heating, the increase in self-heating during verification is not excessively high [12]. However, thermal drift may introduce a thermal error to the MT and it should be modelled.

Currently, the main manners to model the thermal drift of the machine are the thermal error prediction using finite element analysis on all MTs components, and the modelling of the thermal influences on the MT structural elements that control the MT movements. The first is the most accurate manner; however, substantial information about the machine design and the thermal expansion coefficients of all of its components is required [4,15,16]. In addition, its computational cost is high and, typically, the system provider does not have all the required information. The latter (i.e., the thermal drift error model) is not as accurate as the finite element analysis; nevertheless, it is more flexible [17].

To take into consideration the influence of temperature on MT, thermal variations should be measured along verification. As temperature is different on each MT element, internal or surface temperature sensors are placed along machine tool structural elements, capturing data every few seconds. Nonetheless, the data-recording obtained from each sensor should be linked with the MT conditions when every verification point is measured. This depends on the verification conditions, and the verification time for the LT (T_v_) is calculated using Equation (4).

(4) $$T_{v}=\;\frac{L_{T}}{F}+\;T_{s}\left(n-1\right),$$

where L_T_ represents the distance travelled by the machine, F represents the feeding rate, T_s_ represents the time to stop the MT, and n represents the number of measured points. This way, the temperature of each structural element that defines the MT kinematic model, when a verification point is measured, is known and can be introduced in Equations (7–9,11–13,15–20). This is explained in the following paragraphs.

To model the temperature variations on the MT during the verification process (with a reference temperature of 20 °C), it was considered that the influence of the thermal variations is proportional to the distance. Hence, the temperature of a point between two sensors on the same axis is linearly interpolated.

When this assumption is made, the kinematic model of the MT with an XFYZ configuration can be defined as in Equation (2), where the offset of the tool $\bar{T}$ is calculated from:

(5) $$\bar{T}=\left(\begin{array}{c} x_{t}\\ y_{t}\\ z_{t} \end{array}\right).$$

$\bar{R_{k}}$ represents the rotational errors matrix of axis k with k = x, y, z:

(6) $$\bar{R_{\left(k\right)}}=\left(\begin{array}{ccc} 1 & -\varepsilon_{z}\left(k\right) & \varepsilon_{y}\left(k\right)\\ \varepsilon_{z}\left(k\right) & 1 & -\varepsilon_{x}\left(k\right)\\ -\varepsilon_{y}\left(k\right) & \varepsilon_{x}\left(k\right) & 1 \end{array}\right),$$

where $\varepsilon_{x}\left(k\right)$, $\varepsilon_{y}\left(k\right)$, and $\varepsilon_{z}\left(k\right)$ are the three rotational errors of axis k = x, y, z. $\bar{X}$, $\bar{Y}$, and $\bar{Z}$ represent the translational error vector in the x-, y-, and z-axis of the milling machine, respectively.

(7) $$\bar{X}=\left(\begin{array}{c} -\left(x+x\cdot \alpha_{x}\cdot \left(T_{i}-T_{20}\right)\right)+\delta_{x}\left(x\right)\\ \delta_{y}\left(x\right)\\ \delta_{z}\left(x\right) \end{array}\right),$$

(8) $$\bar{Y}=\left(\begin{array}{c} \delta_{x}\left(y\right)-y\cdot s_{xy}\\ \left(y+y\cdot \alpha_{y}\cdot \left(T_{i}-T_{20}\right)\right)+\delta_{y}\left(y\right)\\ \delta_{z}\left(y\right) \end{array}\right),$$

(9) $$\bar{Z}=\left(\begin{array}{c} \delta_{x}\left(z\right)-z\cdot s_{xz}\\ \delta_{y}\left(z\right)-z\cdot s_{yz}\\ \left(z+z\cdot \alpha_{z}\cdot \left(T_{i}-T_{20}\right)\right)+\delta_{z}\left(z\right) \end{array}\right),$$

where $\delta_{k}\left(k\right)$ is the position error of axis k = x, y, z, $\delta_{k}\left(j\right)$ (with k ≠ j being the straightness error in the k direction) and $s_{xy},\;s_{xz},\;\mathrm{and}\;s_{yz}$ are the squareness errors. $\alpha_{i=x,y,z}$ is the coefficient of thermal expansion from axes x, y, and z, respectively. $T_{i}$ represents the value of the temperatures related to axis i = x, y, z, which is related to sensor values. T_20_ represents a reference temperature at 20 °C.

The coefficient of thermal expansion, $\alpha_{i=x,y,z}$, has an associated uncertainty value. It is recommended to use a minimum value of 10% of the nominal value, never lower than 0.002 µm/mm k. This value becomes a standard deviation [14] and is calculated as:

(10) u(αi=x,y,z)= 0.00223=0.0006 μmmm·K.

Hence, the uncertainty of the thermal expansion coefficient may be included in Equations (7)–(9) of the MT kinematic model. Equations (11)–(13) yield the results.

(11) $$\bar{X}=\left(\begin{array}{c} -\left(x+x\cdot \left(\alpha_{x}+u\left(\alpha_{x}\right)\right)\cdot \left(T_{i}-T_{20}\right)\right)+\delta_{x}\left(x\right)\\ \delta_{y}\left(x\right)\\ \delta_{z}\left(x\right) \end{array}\right),$$

(12) $$\bar{Y}=\left(\begin{array}{c} \delta_{x}\left(y\right)-y\cdot s_{xy}\\ \left(y+y\cdot \left(\alpha_{y}+u\left(\alpha_{y}\right)\right)\cdot \left(T_{i}-T_{20}\right)\right)+\delta_{y}\left(y\right)\\ \delta_{z}\left(y\right) \end{array}\right),$$

(13) $$\bar{Z}=\left(\begin{array}{c} \delta_{x}\left(z\right)-z\cdot s_{xz}\\ \delta_{y}\left(z\right)-z\cdot s_{yz}\\ \left(z+z\cdot (\alpha_{z}+u\left(\alpha_{z}\right)\right)\cdot \left(T_{i}-T_{20}\right))+\delta_{z}\left(z\right) \end{array}\right).$$

Another manner to determine the influence of the environmental variation has been described in the ISO/TR230-9 [14] via Equation (14) using the E_VE_ drift value. This can be calculated by considering the change in the results at the most extreme position during the time necessary to perform the test.

(14) $$u_{EVE}=\;\frac{E_{VE}}{2\sqrt{3}}$$

The E_VE_ value should be obtained without the movement of the axes. Therefore, it cannot be used directly in the kinematic model of the machine; however, it might be used to analyze previous variations of the LT measurement as a result of its internal heating [18].

If the number of variables is sufficiently high, the central limit theorem states that the distribution of the sum of the variables is asymptotically normal and independent of the distributions of the individual variables. Therefore, Equations (11) through (13) are modified to become Equations (15) through (17), where β_i_ = x, y, z is the backlash error of axes x, y, and z, respectively, R_i_ = x, y, z is the axes repeatability error, and C_i_ = x, y, z is the control error of axes x, y, and z, respectively, assuming a joint influence defined as a normal distribution.

(15) $$\bar{X}=\left(\begin{array}{c} -\left(x+\beta_{x}+u\;\left(\beta_{x},R_{x},C_{x}\right)\right)\cdot \left(1+\left(\alpha_{x}+u\left(\alpha_{x}\right)\right)\cdot \left(T_{i}-T_{20}\right)\right)+\delta_{x}\left(x\right)\\ \delta_{y}\left(x\right)\\ \delta_{z}\left(x\right) \end{array}\right),$$

(16) $$\bar{Y}=\left(\begin{array}{c} \delta_{x}\left(y\right)-\left(y+\beta_{y}+u\;\left(\beta_{y},R_{y},C_{y}\right)\right)\cdot \left(1+\left(\alpha_{y}+u\left(\alpha_{y}\right)\right)\cdot \left(T_{i}-T_{20}\right)\right)\cdot s_{xy}\\ \left(y+\beta_{y}+u\;\left(\beta_{y},R_{y},C_{y}\right)\right)\cdot \left(1+\left(\alpha_{y}+u\left(\alpha_{y}\right)\right)\cdot \left(T_{i}-T_{20}\right)\right)+\delta_{y}\left(y\right)\\ \delta_{z}\left(y\right) \end{array}\right),$$

(17) $$\bar{Z}=\left(\begin{array}{c} \delta_{x}\left(z\right)-\left(z+\beta_{z}+u\;\left(\beta_{z},R_{z},C_{z}\right)\right)\cdot \left(1+(\alpha_{z}+u\left(\alpha_{z}\right)\right)\left(T_{i}-T_{20}\right))\cdot s_{xz}\\ \delta_{y}\left(z\right)-\left(z+\beta_{z}+u\;\left(\beta_{z},R_{z},C_{z}\right)\right)\cdot \left(1+(\alpha_{z}+u\left(\alpha_{z}\right)\right)\left(T_{i}-T_{20}\right))\cdot s_{yz}\\ \left(z+\beta_{z}+u\;\left(\beta_{z},R_{z},C_{z}\right)\right)\cdot \left(1+(\alpha_{z}+u\left(\alpha_{z}\right)\right)\cdot \left(T_{i}-T_{20}\right))+\delta_{z}\left(z\right) \end{array}\right).$$

Finally, the geometric errors are an additional source of uncertainty provided by the MT. This has been modelled and studied in depth as both a normal and a beta distribution [19]. MT errors can be interpreted as a constant value plus a random one, which provides a probabilistic distribution. Therefore, all constant or deterministic errors, i.e., $\varepsilon_{x}\left(k\right),$ $\varepsilon_{y}\left(k\right),$ $\varepsilon_{z}\left(k\right),$ $\delta_{k}\left(k\right),$ $\delta_{k}\left(j\right),\;\varepsilon_{xy},\;\varepsilon_{xz},\;\mathrm{and}\;\varepsilon_{yz}$, presented in the kinematic model of the machine, have an associated uncertainty, i.e., $u(\varepsilon_{x,k})$, $u\left(\varepsilon_{yk}\right),\;u\left(\varepsilon_{zk}\right)$, $u(\delta_{kk}),$ $u(\delta_{kj})$, $u(\varepsilon_{xy})$, $u(\varepsilon_{xz})$, and $u\left(\varepsilon_{yz}\right)$. Equations (18) through (20) show how Equations (15) through (17) are modified. Similar effects are also presented in Equation (6).

(18) $$\bar{X}=\left(\begin{array}{c} -\left(x+\beta_{x}+u\;\left(\beta_{x},R_{x},C_{x}\right)\right)\cdot \left(1+\left(\alpha_{x}+u\left(\alpha_{x}\right)\right)\cdot \left(T_{i}-T_{20}\right)\right)+\delta_{x}\left(x\right)+u\left(\delta_{x}\left(x\right)\right)\\ \delta_{y}\left(x\right)+u\left(\delta_{y}\left(x\right)\right)\\ \delta_{z}\left(x\right)+u\left(\delta_{z}\left(x\right)\right) \end{array}\right),$$

(19) $$\bar{Y}=\left(\begin{array}{c} \delta_{x}\left(y\right)+u\left(\delta_{x}\left(y\right)\right)-\left(y+\beta_{y}+u\;\left(\beta_{y},R_{y},C_{y}\right)\right)\cdot \left(1+\left(\alpha_{y}+u\left(\alpha_{y}\right)\right)\cdot \left(T_{i}-T_{20}\right)\right)\cdot \left(s_{xy}+u\left(s_{xy}\right)\right)\\ \left(y+\beta_{y}+u\;\left(\beta_{y},R_{y},C_{y}\right)\right)\cdot \left(1+\left(\alpha_{y}+u\left(\alpha_{y}\right)\right)\cdot \left(T_{i}-T_{20}\right)\right)+\delta_{y}\left(y\right)+u\left(\delta_{y}\left(y\right)\right)\\ \delta_{z}\left(y\right)+u\left(\delta_{z}\left(y\right)\right) \end{array}\right),$$

(20) $$\bar{Z}=\left(\begin{array}{c} \delta_{x}\left(z\right)+u\left(\delta_{x}\left(z\right)\right)-\left(z+\beta_{z}+u\;\left(\beta_{z},R_{z},C_{z}\right)\right)\cdot \left(1+(\alpha_{z}+u\left(\alpha_{z}\right)\right)\left(T_{i}-T_{20}\right))\cdot (s_{xz}+u\left(s_{xz}\right))\\ \delta_{y}\left(z\right)+u\left(\delta_{y}\left(z\right)\right)-\left(z+\beta_{z}+u\;\left(\beta_{z},R_{z},C_{z}\right)\right)\cdot \left(1+(\alpha_{z}+u\left(\alpha_{z}\right)\right)\left(T_{i}-T_{20}\right))\cdot (s_{yz}+u\left(s_{yz}\right))\\ \left(z+\beta_{z}+u\;\left(\beta_{z},R_{z},C_{z}\right)\right)\cdot \left(1+(\alpha_{z}+u\left(\alpha_{z}\right)\right)\cdot \left(T_{i}-T_{20}\right))+\delta_{z}\left(z\right)+u\left(\delta_{z}\left(z\right)\right) \end{array}\right).$$

#### 2.2.2. Uncertainty Related to the Measurement System

Several researchers studied laser tracker error sources [18,20,21]. Gallagher et al. [20] divided error sources as angular encoders, tracking, and assembly errors. Meanwhile, Knapp [21] divided errors sources as environmental errors, data captures, and simplifications. If all these errors sources are analyzed together, two principal groups can be done.

The first one is related to systematic errors, such as environmental conditions. Pressure, temperature, and humidity produce a variation of the refraction index of the air, which provides an error in the laser wavelength estimation, which affects the measurement distance. However, these errors present a systematic behavior that can be analytically compensated due to a meteorological LT station and control. Other systematic errors that cannot be compensated by LT control, such as warm-up time, can be characterized and its influence reduced [18].

The second one consists of random error sources, such as uncertainty from radical and angular encoders. The influence of these errors sources is defined as measurement noise. In this way, manufacturers provide specifications for their LTs, and their accuracy is combined based on the ISO DIS 10360-10 and ASME B 89.4.19-2005 standards [22,23]. It is the joint effect of the uncertainties of the LT components and depends on the employed encoders and sensors.

**Figure 4:**
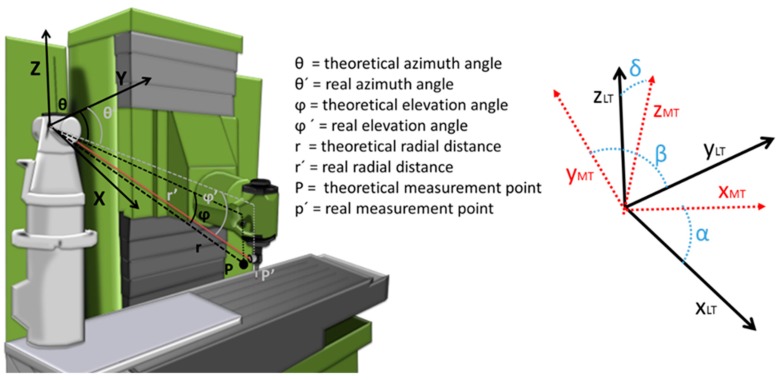
LT measurement uncertainty and location parameters.

As shown in the equation of movement of the MT (Equation (2)), the influence of the relation between the LT and the MT on the verification (Figure 4) is derived from a rotation matrix $\bar{R}\left(lt\right)$ defined by the Euler angles, α, β, and δ, and a translational vector, $\bar{T_{LT}}$. This vector and matrix are calculated as shown in Equations (21) and (22), respectively:

(21) $$\bar{T_{LT}}=\left[D,L,H\right],$$

(22) $$\bar{R_{LT}}=(\begin{array}{ccc} \cos\left(\beta \right)\cos\left(\delta \right) & -\cos\left(\beta \right)\sin\left(\delta \right) & \sin\left(\beta \right)\\ \cos\left(\alpha \right)\sin\left(\delta \right)+\sin\left(\alpha \right)\sin\left(\beta \right)\cos\left(\delta \right) & \cos\left(\alpha \right)\cos\left(\delta \right)-\sin\left(\alpha \right)\sin\left(\beta \right)\sin\left(\delta \right) & -\sin\left(\alpha \right)\cos\left(\beta \right)\\ \sin\left(\alpha \right)\sin\left(\delta \right)-\cos\left(\alpha \right)\sin\left(\beta \right)\cos\left(\delta \right) & \sin\left(\alpha \right)\cos\left(\delta \right)+\cos\left(\alpha \right)\sin\left(\beta \right)\sin\left(\delta \right) & \cos\left(\alpha \right)\cos\left(\beta \right) \end{array}).$$

Influence of systematic errors can be reduced, but measured points are affected by LT measurement uncertainties originating from angular encoders and the radial distance, as per Equations (23), random errors (Figure 4). This equation links the data from the encoders and the radial distance with their uncertainty; the uncertainty is provided by means of a point measured in Cartesian coordinates:

(23) $$\left[\begin{array}{c} u_{x}^{2}\\ u_{y}^{2}\\ u_{z}^{2} \end{array}\right]=\left[\begin{array}{ccc} \sin^{2}\theta \cdot \cos^{2}\varphi & r^{2}\cdot \cos^{2}\theta \cdot \cos^{2}\varphi & r^{2}\cdot \sin^{2}\theta \cdot \sin^{2}\varphi \\ \sin^{2}\theta \cdot \sin^{2}\varphi & r^{2}\cdot \cos^{2}\theta \cdot \sin^{2}\varphi & r^{2}\cdot \sin^{2}\theta \cdot \cos^{2}\varphi \\ \cos^{2}\theta & r^{2}\cdot \sin^{2}\theta & 0 \end{array}\right]\cdot \left[\begin{array}{c} u_{r}^{2}\\ u_{\theta }^{2}\\ u_{\varphi }^{2} \end{array}\right].$$

In this case, *r* represents the radial measured distance, $u_{r}$ the radial uncertainty, *θ* the azimuth angle, $u_{\theta }$ the azimuth angle uncertainty, φ the polar angle, and $u_{\varphi }$ the polar angle uncertainty. The uncertainty of a measured point is presented in Equation (24).

(24) $$u_{p}^{2}=u_{x}^{2}+u_{y}^{2}+u_{z}^{2}.$$

Moreover, a retro-reflector as an element of a measurement system is a source of uncertainty [19]. The LT laser beam should strike at the center of a reflector. If not, the influence of the retro-reflector uncertainty ($u_{SMR}$) must be modelled as a normal distribution and included in the equations.

The employed work procedure is an additional uncertainty source; however, it cannot be modelled. Moreover, metrology software allows one to define the measurements that modify the frequency and samples per point; the software provides an uncertainty for each measured point, i.e., $u_{MS,x\;,}u_{MS,y\;},u_{MS,z}$.

#### 2.2.3. Uncertainty Related to the Measurement Strategy

As has been described in the previous section, the LT should be located to improve data accuracy. However, if actual verification is conducted, the LT position is obtained using a least-squares adjustment between the MT nominal coordinates and the actual points measured using LT. As the LT-measured points are affected by different sources of error (such as the measured noise), the rotation matrix $\bar{R}\left(lt\right)$ and the translational vector $\bar{T_{LT}}$ are affected as well. Moreover, the least-squares adjustment is a mathematical tool that minimizes the difference between points, producing its own uncertainty. Thus, random elements are added to the $\bar{R}\left(lt\right)$ and $\bar{T_{LT}}$ components, following a PDF (Equations (25)–(26)).

(25) $$\bar{R}\left(lt\right)=\bar{R}{\left(lt\right)}_{nominal}+\bar{U_{Rlt}}$$

(26) $$\bar{T_{LT}}=\bar{T_{LT,\;nominal}}+\bar{U_{Tlt}}$$

The other source of error related to the measurement strategy is the use of multilateration to improve data accuracy. Studies have shown different results depending on the LT and the employed measurement technique, the measurement mode (IFM—Interferometer or ADM—Absolute Distance Measurement), and the spatial angle between the LT locations. Multilateration prevents angular measurement noise and increases the radial one [24]; however, its uncertainty has not yet been modelled as a PDF related to spatial angles between LTs beams. Due to MT characteristic, tests presented in Section 3 were not carried out based on the multilateration technique, they were done on a singular LT location.

#### 2.2.4. Uncertainty Related to the Optimization Strategy

**Figure 5:**
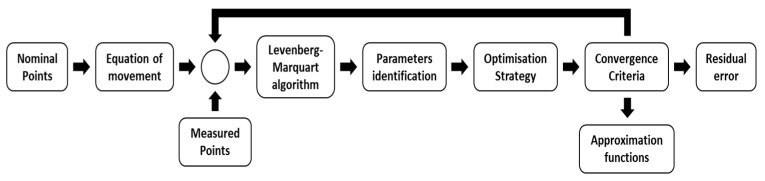
Characterization Procedure Scheme.

The optimization strategy, convergence criteria, and phase optimization (Figure 5) cannot be modelled as previous uncertainty sources; nonetheless, they affect the MT accuracy depending on its configuration and verification design [24]. Therefore, if these are changed, the MT capacity will be modified.

Approximation functions that characterize MT geometric errors may be obtained, thus minimizing the difference between the real and the nominal points. The difference becomes minimized using an iterative process of parameter identification through the MT equation of movements. Therefore, the objective function is the difference between pairs of points (Equation (27)).

(27) $$v_{e,LT}=\;\frac{\sum_{i=1}^{n}\sqrt{{\left(x_{i,n}-x_{i,r}\right)}^{2}+{\left(y_{i,n}-y_{i,r}\right)}^{2}+{\left(z_{i,n}-z_{i,r}\right)}^{2}}}{n}$$

**Figure 6:**
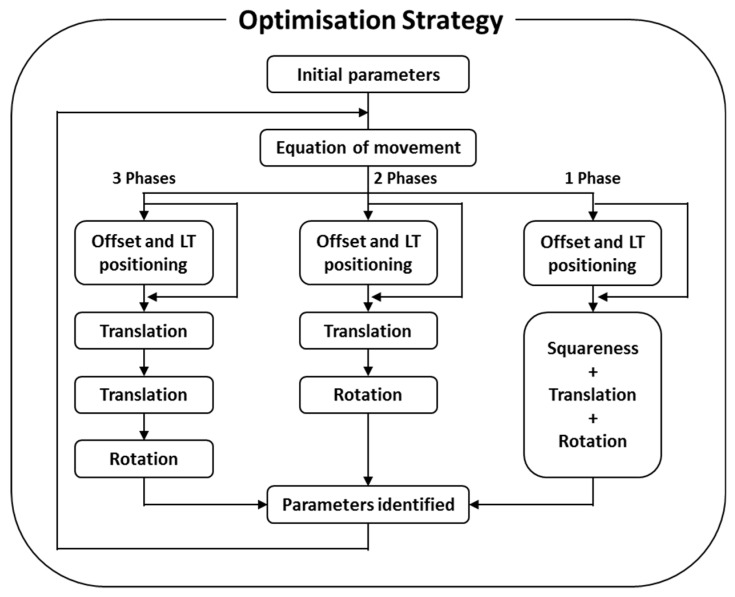
Optimization strategy scheme.

The residual error after verification will depend on the verification design, i.e., the identification method, the approximation functions used, the optimization strategy, the defined initial parameters, and the employed convergence criteria (Figure 6).

The adequacy of the optimization strategy in the verification of the MT configuration has been studied in past research works [25]. Considering this, the best optimization strategy for the XFYZ MT configuration was implemented and was not modified in the verification process of the tests presented in this paper (Section 4) as a prior step for the estimation of the verification uncertainty.

### 2.3. Monte Carlo Method for Uncertainty Evaluation

Every measurement should be provided with an estimation of its measurement uncertainty. Currently, a standard guide exists for the estimation of the measurement uncertainty, namely the “Guide to the expression of uncertainty in measurement” (GUM) [26]. However, it should not be used for non-linear models, such as the MT studied in this paper. Supplement 1 of GUM recommends the use of the Monte Carlo method (MCM) in such cases.

**Figure 7:**
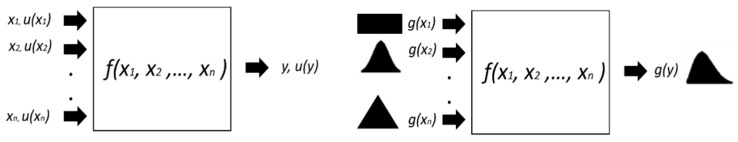
Propagation of uncertainties based on GUM (Guide to the expression of uncertainty in measurement) (**left**); propagation of distributions based on the Monte Carlo method (MCM) (**right**).

The Monte Carlo method employs a large number of samples generated according to different propagation probabilistic functions (Figure 7) to obtain the final uncertainty distribution through the measurement equation, i.e., Equation (2).

To estimate the volumetric verification uncertainty using MCM, the first step is the definition of statistical distributions of measured-input quantities. The principal ones are divided into MT errors, measurement strategy errors, measurement system errors, and optimization strategy, as shown in Figure 3. All of them have been presented and analyzed in depth in Section 2.2. Secondly, the mathematical model that describes the process must be defined; in this case, the movement equation provided by the MT kinematic chain affected by the input uncertainties has been presented in Section 2.2. Next, using verification conditions, tests were conducted for the measurement data; moreover, the PDFs for the input quantities were estimated. At this stage, the verification tests were created and the MCM simulation was set up and run. When all tests had been completed, the last step was to summarize and present the results.

**Figure 8:**
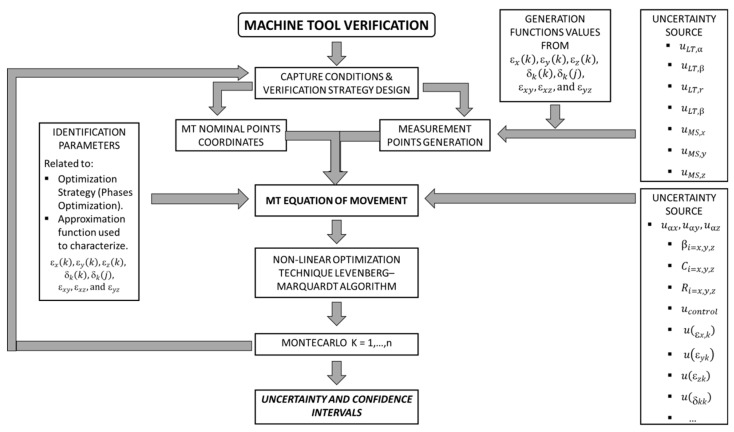
Uncertainty evaluation and confidence intervals of the parameters.

Several researchers have merely taken the first step towards studying MTs, considering different distributions functions, thermal variations, or measurement noise [2,3,4,23] Figure 8 shows the working principle of the development of the software used for the estimation of the verification uncertainty of an MT using LT as measurement system through volumetric verification.

## 3. Simulations Results

To use an MT as a measurement system with traceability, it needs to improve its positioning accuracy. It will depend on the PDF of its final verification volumetric error, which should be obtained to estimate its uncertainty.

To study the influence of LT measurement uncertainty in LT positioning and verification uncertainty, MCM was used. The best LT position related to measurement uncertainty was obtained using Equation (24). The values of $u_{\theta }$ and $u_{\varphi }$ were derived from the normal probability distribution, i.e., µ = 20 µrad and σ = 3 µrad, and $u_{d}$ was defined as 4 µm ± 0.8 µm/m for radial coordinate. Therefore, each point to measure will yield different uncertainty values in each test. To achieve this, simulation tests were conducted in a three-axis MT with an XFYZ configuration.

The Monte Carlo method tests were defined as follows:

- MT workspace to verify: 800 mm ≤ X ≤ 1200 mm, 100 mm ≤ Y ≤ 500 mm, and 200 mm ≤ Z ≤ 400 mm.
- The verification mesh contained 75 verification points with intervals of 100 mm in all axes.
- Available space to locate LT: −2000 mm ≤ D ≤ −500 mm, 350 mm ≤ H ≤ 2000 mm, −2000 mm ≤ L ≤ 2000 mm.
- LT measurement range characteristic: 0.5 m ≤ r ≤ 15 m, −45° ≤ θ ≤ 45°; −235° ≤ φ ≤ 235°.
- LT could not be located inside the MT workspace or body for verification.
- The number of Monte Carlo tests was 100.000 in the X-axis and Y-axis directions.

The main objective of these tests was not only to obtain the best LT location, but also to be able to associate the LT position with the influence of LT measurement uncertainty in the verification mesh. In this manner, although measurement uncertainty would be smaller than the maximum error introduced in most of the verification points, an approximate value below which the MT measurement/position accuracy is within the uncertainty zone (Figure 1) would be obtained.

**Figure 9:**
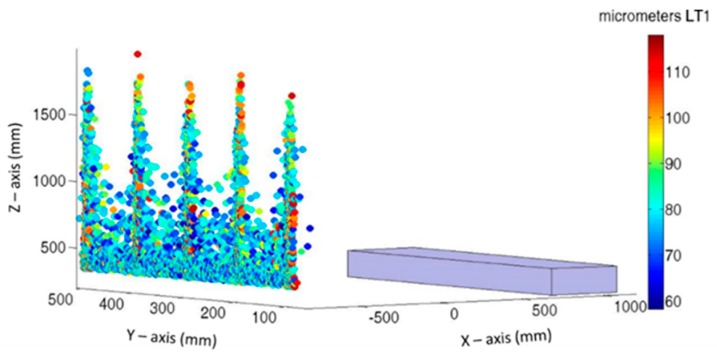
Best LT position and maximum error obtained on each Monte Carlo test: Y-axis direction.

**Figure 10:**
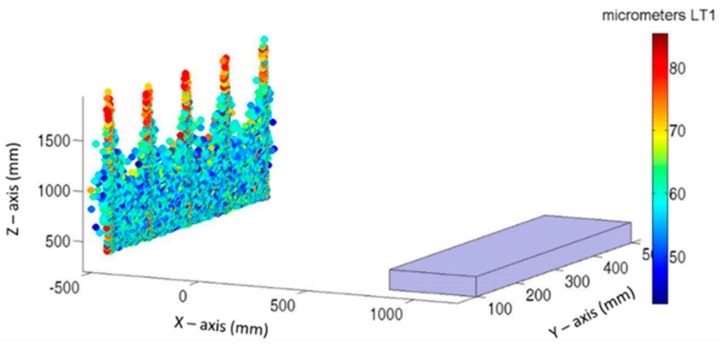
Best LT position and maximum error obtained on each Monte Carlo test: X-axis direction.

Figure 9 and Figure 10 show the LT location and the maximum error introduced to the verification mesh for each Monte Carlo test where LTs are located along the Y-axis and X-axis of the MT, respectively. Each point represents the best location to a definite test, and its color represents the maximum error introduced on it by LT. This value is obtained as the maximum absolute value of Equation (1).

If both figures are compared, there is a central zone with Z values lower than 700 mm in Figure 9 and 1100 mm in Figure 10, where there is a higher concentration of lower maximum errors. In the same manner, there are five columns that rise in the Z-axis, with a higher concentration of upper values. In both cases, these columns are located in the same planes (Y and X) as that of the verification points.

However, certain differences may be observed between both figures. Lower values in Figure 9 lie between 60 and 80 µm; the lowest values of Figure 10 lie between 40 and 60 µm. This means that the influence of noise has been reduced by approximately 30%. In the same manner, the maximum values in Figure 9 are approximately 30 µm higher than the maximum values in Figure 10.

**Figure 11:**
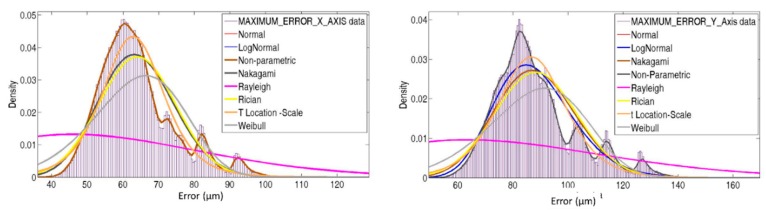
Maximum error for each best LT position.

The color maps presented in Figure 9 and Figure 10 provide an efficient manner to observe the spatial areas where LT should be placed. However, to identify which frequency obtained the highest values and which PDFs best characterized the behavior, a histogram of both cases is presented in Figure 11.

Figure 11 presents the $u_{p}$ values obtained from Equation (24); hence, no negative values exist, in addition to the LT position and the maximum error introduced for each one. Although measurement uncertainty from radial and angular encoders has been defined as a normal distribution, none of the presented PDFs describes the real distribution of maximum errors properly.

To study the influence of LT measurement uncertainty on the verification uncertainty, the verification of the same mesh was simulated 1000 times. The working principle was presented in Section 2, and the parameters required were:

- Equation of movement of the MT and its kinematic model (Equation (2)).
- Verification mesh of points (in this case, the same used to determine the LT position).
- Laser tracker position: d = –563.52 mm, l = 194.37 mm, h = 677.78 mm, α = 0.0339°, β = 0.0343°, and δ = 59.4652°, obtained in LT position from previous tests.
- Generation functions that characterize geometrics of the MT were obtained from real verification.
- Optimization strategy. To identify the geometric errors of the MT, a one phase optimization procedure is used as is shown in Figure 6. From information of a single LT, the influence of squareness, translation, and rotation error are considered together.

**Figure 12:**
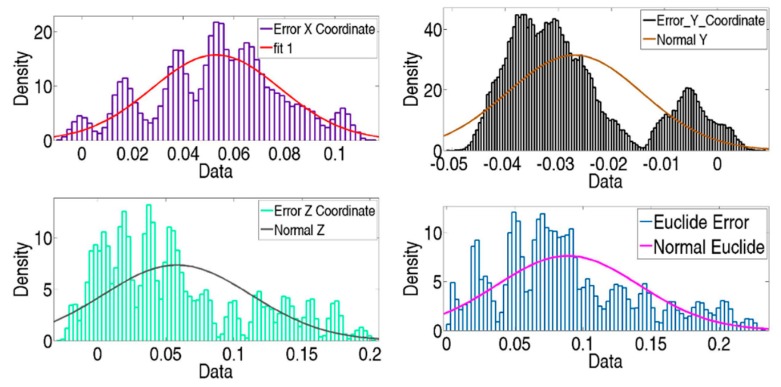
Initial error of MT verification points of 1000 tests.

Figure 12 shows verification point errors in the X, Y, and Z axes in the LT coordinate system. These errors are the joint influence of its errors and LT noise. Although the probability distribution of LT components is normal, the joint influence of geometrical and measurement error cannot fit a normal distribution.

**Figure 13:**
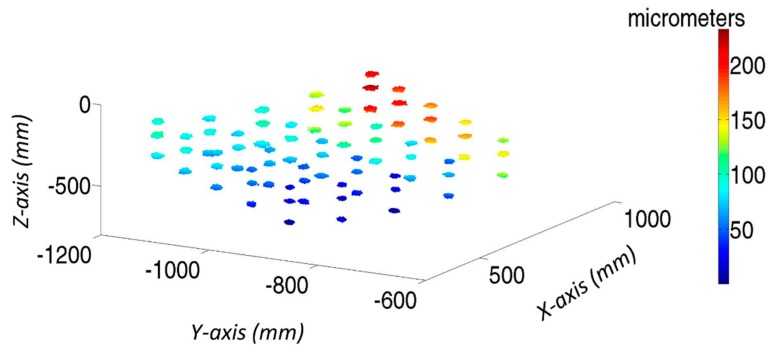
Initial color map of verification points of 1000 tests on laser tracker coordinate system.

Figure 13 analyses the initial error distribution along the verification mesh (Equation (1)). For each verification point, there are 1000 samples affected by the same geometric error; however, with a different LT noise, there is a cloud of measured points around nominal positions. It can be observed that geometric errors have increased from the reference point, in this case the corner of the cube, following a systematic behavior. So, the main aim of volumetric verification is to compensate for this error influence on MT accuracy. Moreover, there are no strong variations in the color of each point cloud; hence, the influence of geometric errors is greater than the measurement noise.

**Figure 14:**
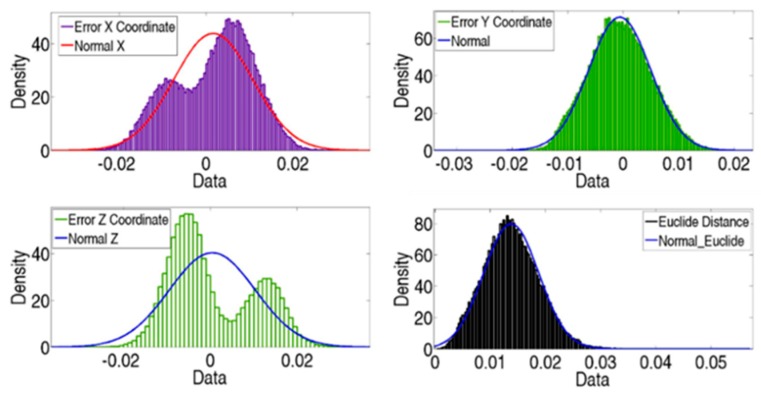
Final error of MT verification points of 1000 tests in total.

Figure 14 shows the final errors in the verification points of all simulated tests. If we compare it with Figure 12, it can be seen that the final error distribution presents a normal distribution, particularly the final error in the distance and the error in the Y coordinate.

**Figure 15:**
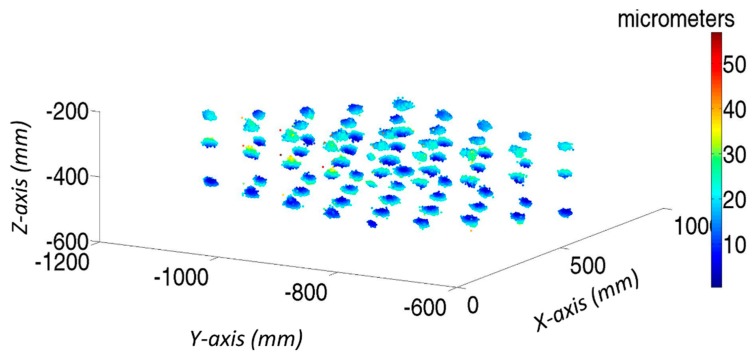
Final color map of verification points of 1000 tests on laser tracker coordinate system.

Figure 15 shows the final residual error in the verification points of all simulated tests, when the influence of geometric errors is compensated. A comparison of Figure 13 and Figure 15 shows that the errors have been greatly minimized. Moreover, the color of the cloud of each verification point is not homogeneous. Although their real geometric errors are the same, each test provides a different geometric error approximation function for each error. This is because of the effects of the LT measurement noise on the approximation functions and therefore on the final positioning of MT accuracy.

The maximum final error after verification is approximately 50 µm, as shown in Figure 15. If compared with the error introduced by the LT measurement noise at its location (Figure 10), the error has a similar value. Hence, the verification uncertainty of the MT is 50 µm, although the final mean volumetric error of the verification tests is a normal distribution with $\bar{X}$ 13.77 µm and σ = 5.02 µm.

## 4. Conclusions

To use an MT as a measurement system in a process with traceability requires the improvement of its positioning accuracy and the determination of its measurement uncertainty. The measurement uncertainty of the MT will be equal or higher than the verification uncertainty.

Main uncertainty sources related to MT verification can be divided into four groups: the MT, measurement system, measurement strategy, and optimization strategy. Uncertainty from the MT and the measurement systems affected nominal and actual measured points. The remaining uncertainty sources did not cause physical consequences; however, they affected the resolution of mathematical errors. We have developed a new kinematic model where the influences of these sources of uncertainty were considered via the modelling of their behavior.

As the developed kinematic model had a non-linear behavior, GUM could not be implemented. Instead, the Monte Carlo method was used to estimate verification uncertainty. The measurement noise from LT was modelled as a normal probability distribution; however, simulated tests demonstrated that its influence on the verification points may not be properly described by any of the presented PDFs used to model the real distribution of maximum errors. A suitable LT location along the MT workspace would provide an average maximum error reduction of approximately 30%.

With the LT at this suitable area, tests showed that the initial-error behavior cannot be modelled as a normal distribution, neither in distance nor in any of the directions of the axes of the machine. Nevertheless, there is a normal cloud of points around nominal positions, i.e., the noise measurements. For each test, different geometric approximation functions were employed with different residual errors. These had a normal behavior, which represented the uncertainty verification; it could be modelled as a normal distribution with $\bar{X}$ = 13.77 µm and *σ* = 5.02 µm. This means that with a confidence level of 95%, the uncertainty value would be lower than 23.81 µm. In this manner, falsely accepted and false-rejection zones would be identified. However, in a few tests, certain verification points presented a maximum position error of approximately 50 µm, which was similar to the maximum error introduced in the LT position tests. Although the error was smaller than 50 µm in most tests, accuracy greater than this value cannot be ensured.
